# Editorials

**Published:** 1894-01

**Authors:** 


### HOMŒOPATHIC PHYSICIAN,

A MONTHLY JOURNAL OF

HOMŒOPATHIC MATERIA MEDICA AND CLINICAL MEDICINE.

"If our school ever gives up the strict inductive method of Hahnemann, we are lost, and deserve only to be mentioned as a caricature in the history of medicine."—CONSTANTINE HERING.

Vol. XIV. JANUARY, 1894. No. 1.

### EDITORIALS.

APPEAL FOR PAYMENT.—In the December number a strong appeal was made to subscribers for the payment of their dues. Whilst not every one has responded to this appeal yet a considerable number have complied with our urgent demands, and as a result our treasury has a little more cash than when the December number was issued, and we are enabled to go to press as usual and even to continue the publication of Wells on Intermittent Fever. Accordingly another fascicle appears in this number.

The amount of money received is not enough, however, and we again must ask the subscribers to pay up their arrears of dues that we may go on with the work to the end of the year without failure.

For the response already made to the December editorial, we return our warmest thanks. It is all the more highly appreciated in view of the general depression in business affairs and the great reduction in the amount of money in circulation.

Most of the subscribers have been exceedingly loyal to the journal, appreciating the efforts made by its editor to keep to the standard of pure Homœopathy and to the straight line of truth.

For the new year we ask a continuance of the excellent contributions to the pages of THE HOMŒOPATHIC PHYSICIAN which have graced them in the past.

The distinguished authors who have written articles that have been warmly received by the profession can render the cause of medicine no greater service than by continuing their writings, and thus sustaining the editor in his own efforts for the benefit of the cause.

---

ISOPATHY.—The excellent article on Tuberculinum by Dr. Horace P. Holmes in this number has aroused in the editor some thoughts upon that curious perversion of Homœopathy called Isopathy. It seems surprising that any one who has once been enlightened by the revelations of Homœopathy, and has the teachings of the old school of medicine to compare them with, should be attracted by the formula of Isopathy.

It would seem almost superfluous to direct the attention of any of our readers to the history of the old school of medicine. Yet that appears to be actually necessary for those who believe in Isopathy.

The one lesson to be learned by a survey of old school history is the continual speculation by the doctors upon the cause and phenomena of disease, the crystallizing of these speculations into theories more or less at variance with the facts, and the treating of the patients upon a "rational basis" which is only the logical sequence of the theories. The attention of the theorist being riveted to the contemplation of his theories, he is but feebly, or not at all, aware of what is perfectly patent to the bystander not burdened with these theories—the wide divergence of his measures of treatment from the lines of efficacy and safety. Only when some perfectly obvious catastrophe overtakes him does he consent to change his method. That means, of course, the substitution of a fresh theory and the repetition of the old errors in a new form. Now, what Hahnemann did was to follow the example of the brilliant physicists who were contemporaneous with him. That was to form no previous theories, but, casting all theories, notions, and opinions behind them, to resort to careful, thoughtful experiment, and deduce from the experiment a view more or less symmetrical from the results of the experiment, the view being amplified or corrected by further experiments as needed. This is the inductive method. The results of such methods of inquiry are always surprising, for they are always widely at variance with the preconceived view. Consequently, when Hahnemann announced his discovery, it was diametrically opposed to everything known or believed, and to what is called common sense. Nevertheless, there it stands, and the ocean of criticism and opposition rolls against it only to surge back again, because it is beating against a rock.

Now, when any one advocates Isopathy, he is advocating a theory. He is indulging in speculation; he is assuming things which he does not know and has not proved; he is simply returning to the field of the old school of medicine, to follow in their paths and repeat their meanderings.

If the product of a disease will cure that disease, which product shall we select? What means have we for deciding what one should be used? If it be tuberculosis, why should we select the sputum, which is a complex compound? According to Dr. Holmes, Dr. Swan's preparation was from the sputum and Dr. Burnett's from the lung tissue. Which is right? They are not alike in character, and so there must be other differences in their effect on the system. How shall we decide? The plain path of the inductive method, the *proving* of these compounds on the healthy is rejected and *assumed* to be unnecessary, and in this very assumption the Isopathist unconsciously returns to the speculative methods of all philosophy and scientific inquiry before the inductive method obtained a standing. So, therefore, before we commit ourselves to the advocating of Isopathic methods, we would best inquire into their relationship to all other modes of treatment of the sick, and all other modes of scientific inquiry. If this be done intelligently, we shall not be deceived by any theory, however specious, nor, because cases here and there are actually cured, will our judgment be reversed or confused.

